# 694. Prediction Tool for Infective Endocarditis in Beta-hemolytic Streptococcal Bacteremia

**DOI:** 10.1093/ofid/ofab466.891

**Published:** 2021-12-04

**Authors:** Ryo Hasegawa, Takahiro Matsuo, Osamu Takahashi, Nobuyoshi Mori

**Affiliations:** 1 St Luke's International Hospital, Chuo City, Tokyo, Japan; 2 St. Luke's International Hospital, Tokyo, Japan

## Abstract

**Background:**

Although beta-hemolytic streptococci (BHS) is a rare causative pathogen of infective endocarditis (IE), IE is a serious condition and it is important to predict IE in BHS bacteremia (BHS-IE). The purpose of this study was to develop a predictive score for BHS-IE.

**Methods:**

We conducted a retrospective study comparing the clinical features of BHS-IE and BHS-non infective endocarditis (BHS-nIE) in adult patients with BHS bacteremia at a 520-bed tertiary hospital in Tokyo, Japan from 2004 to 2020. IE was diagnosed according to modified Duke's criteria, and both “Definite” and “Possible” were included. Univariate and multivariable analyses were conducted using logistic regression.

**Results:**

Among 250 patients with BHS bacteremia, 47 (19%) were diagnosed with BHS-IE. The median (IQR) patient age was 71 (59, 84) years and 121 (68%) were male. The proportions of A, B, C/G groups were 14%, 38.4%, and 47.6%, respectively. Five predictors, either independently associated with BHS-IE or clinically relevant, were used to develop the prediction score: C-reactive protein ≥ 10 mg/dl (2 points); Group B Streptococci (1 point); Auscultation of heart murmur (1 point); Platelet count < 150 /µl (1 point); and Hypotension (systolic blood pressure < 90 mmHg or on vasopressor) (1 point). In a receiver operating characteristic analysis, the area under the curve was 0.74 (95% confidence interval [CI]: 0.66 - 0.82). The cut-point was 2. A score ≥2 had a sensitivity of 87% (95%CI: 0.743 - 0.952), a specificity of 37% (95%CI: 0.308 - 0.445), a positive predictive value of 24%, and a negative predictive value of 93%, respectively.

**Conclusion:**

We developed the score to help clinicians rule out IE in BHS bacteremia. Further research is warranted for validation.

**Disclosures:**

**All Authors**: No reported disclosures

